# Supplementary material for: Piezo2 tension sensitivity and its modulation by alternative splicing
Source: bioRxiv. 2026 Feb 17:2026.02.16.706133. Preprint. [Version 1] doi: 10.64898/2026.02.16.706133 (PMC12934605; doi:10.64898/2026.02.16.706133)
Supplement: Supplement 1 [file NIHPP2026.02.16.706133v1-supplement-1.pdf]

bioRxiv preprint doi: https://doi.org/10.64898/2026.02.16.706133; this version posted February 17, 2026. The copyright holder for this preprint (which was not certified by peer review) is the author/funder, who has granted bioRxiv a license to display the preprint in perpetuity. It is made available under aCC-BY 4.0 International license.

Supplemental information

Piezo2 tension sensitivity and its modulation by alternative splicing

Michael Sindoni[1], William Sharp, and Jörg Grandl[1,*]

[1] Department of Neurobiology, Duke University Medical Center, Durham, NC 27710, USA.

*Correspondence: grandl@neuro.duke.edu

bioRxiv preprint doi: https://doi.org/10.64898/2026.02.16.706133; this version posted February 17, 2026. The copyright holder for this preprint (which was not certified by peer review) is the author/funder, who has granted bioRxiv a license to display the preprint in perpetuity. It is made available under aCC-BY 4.0 International license.

Supplemental Figure 1

```
                      TM 1              TM 2                TM 3
mPiezo2   MASEVVCGLIFRLLLPICLAVACAFRYNGLSFVYLIYLLLIPLFSEPTKATMQGHTGRLLQSLCITSLSFLLLHIIFHITLASLEAQHRITPAYNCSTWEKTFRQIGFES   110
hPiezo2   MASEVVCGLIFRLLLPICLAVACAFRYNGLSFVYLIYLLLIPLFSEPTKTTMQGHTGRLLKSLCFISLSFLLLHIIFHITLVSLEAQHRIAPGYNCSTWEKTFRQIGFES   110
          ************************************************* ********** ***  *************** ******** * *****************

                      TM 4
mPiezo2   LKGADAGNGIRVFVPDIGMFIASLTIWLVCRTIVKKPDTEEIAQLNSECENEELAGGEKMDSEEALIYEEDLDGEEGMEGELEESTKLKILRRFASVASKLKEFIGNMIT   220
hPiezo2   LKGADAGNGIRVFVPDIGMFIASLTIWLLCRNIVQKPVTDEAAQSNPEFENEELAEGEKIDSEEALIYEEDFNGGDGVEGELEESTKLKMFRRLASVASKLKEFIGNMIT   220
          **************************** ** ** ** * * ** * * ****** *** ***********  *  * ***********  ** ****************

            TM 5              TM 6                    TM 7
mPiezo2   TAGKVVVTILLGSSGMMLPSLTSAVYFFVFLGLCTWWSWCRTFDPLLFGCLCVLLAIFTAGHLIGLYLYQFQFFQEAVPPNDYYARLFGIKSVIQTDCASTWKIIVNPDL   330
hPiezo2   TAGKVVVTILLGSSGMMLPSLTSSVYFFVFLGLCTWWSWCRTFDPLLFSCLCVLLAIFTAGHLIGLYLYQFQFFQEAVPPNDYYARLFGIKSVIQTDCSSTWKIIVNPDL   330
          *********************** ************************ ************************************************* ***********

               TM 8                                                                Exon 10
mPiezo2   SWYHHANPILLLVMYYTLATLIRIWLQEPLVQEEMAKEDEGALDCSSNQNTAERRRSLWYATQYPTDERKLLSMTQDDYKPSDGLLVTVNGNPVDYHTIHPSLPIENGPA   440
hPiezo2   SWYHHANPILLLVMYYTLATLIRIWLQEPLVQDEGTKEEDKALACSPIQITAGRRRSLWYATHYPTDERKLLSMTQDDYKPSDGLLVTVNGNPVDYHTIHPSLPMENGPG   440
          ******************************** *  **   ** **  * ** ********* ***************************************** ****

                                                              TM 9                       TM 10           TM 11
mPiezo2   KTDLYTTPQYRWEPSEESSEKKEEEEDKREDSEGEGSQEEKRSVRMHAMVAVFQFIMKQSYICALIAMMAWSITYHSWLTFVLLIWSCTLWMIRNRRKYAMISSPFMVVY   550
hPiezo2   KADLYSTPQYRWEPSDESSEKREEEEEEKEEFEEERSREEKRSIKVHAMVSVFQFIMKQSYICALIAMMAWSITYHSWLTFVLLIWSCTLWMIRNRRKYAMISSPFMVVY   550
          * *** ********* ***** ****   *  * * * *****   **** ***********************************************************

                                           TM 12
mPiezo2   ANLLLVLQYIWSFELPEIKKVPGFLEKKEPGELASKILFTITFWLLLRQHLTEQKALREKEALLSEVKIGSQELEEKEDEELQDVQVEGEPTEKEEEEEEEIKEERHEVK   660
hPiezo2   GNLLLILQYIWSFELPEIKKVPGFLEKKEPGELASKILFTITFWLLLRQHLTEQKALQEKEALLSEVKIGSQENEEK-DEELQDIQVEGEPKEEEE---EEAKEEKQERK   656
           **** *************************************************** *************** *** ****** ****** * **   ** ***  * *

                                       TM 13               TM 14                       TM 15
mPiezo2   KEEEEEVEEDDDQDIMKVLGNLVVALFIKYWIYVCGGMFFFVSFEGKIVMYKIIYMVLFLFCVALYQVHYEWWRKILKYFWMSVVIYTMLVLIFIYTYQFENFPGLWQNM   770
hPiezo2   KVEQEEAEEEDEQDIMKVLGNLVVAMFIKYWIYVCGGMFFFVSFEGKIVMYKIIYMVLFLFCVALYQVHYEWWRKILKYFWMSVVIYTMLVLIFIYTYQFENFPGLWQNM   766
          * * ** ** * ************* ************************************************************************************

                             TM 16                                               Exon 18     Exon 19
mPiezo2   TGLKKEKLEDLGLKQFTVAELFTRIFIPTSFLLVCILHLHYFHDRFLELTDLKSIPSKEDNTIYSHAKVNGRVYLIINRIKKKLPIHQNLAHPEGSLPDLAIMNMTASLD   880
hPiezo2   TGLKKEKLEDLGLKQFTVAELFTRIFIPTSFLLVCILHLHYFHDRFLELTDLKSIPSKEDNTIYRHAKVNGRVYLIINSIKKKLPIHQNLAHPEGSLPDLTMMHLTASLE   876
          **************************************************************** ************* *********************  *  ****

                                                                                                  TM 17
mPiezo2   KPEVQKLAESGEERPEECVKKTEKGEAGKDSDESEEEEDEEEESE-EEESSDLRNKWHLVIDRLTVLFLKFLEYFHKLQVFMWWILELHIIKIVSSYIIWVTVKEVSLFN   989
hPiezo2   KPEVRKLAEPGEEKLEGYSEKAQKGDLGKDSEESEEDGEEEEESEEEEETSDLRNKWHLVIDRLTVLFLKFLEYFHKLQVFMWWILELHIIKIVSSYIIWVSVKEVSLFN   986
          **** **** ***  *    *  **  **** ****   ****** *** *************************************************** ********

             TM 18          TM 19    Exon 22                                                                 TM 20
mPiezo2   YVFLISWAFALPYAKLRRAASSVCTVWTCVIIVCKMLYQLQTIKPENFSVNCSLPNENQTNIPLHELNKSLLYSAPVDPTEWVGLRKSSPLLVYLRNNLLMLAILAFEVT   1099
hPiezo2   YVFLISWAFALPYAKLRRLASSVCTVWTCVIIVCKMLYQLQTIKPENFSVNCSLPNENQTNIPFNELNKSLLYSAPIDPTEWVGLRKSSPLLVYLRNNLLMLAILAFEVT   1096
          ****************** ********************************************  *********** *********************************

                                                               TM 21            TM 22                    TM 23
mPiezo2   VYRHQEYYRGRNNLTAPVSKTIFHDITRLHLDDGLINCAKYFVNYFFYKFGLETCFLMSVNVIGQRMDFYAMIHACWLIGVLYRRRRKAIAEVWPKYCCFLACIITFQYF   1209
hPiezo2   IYRHQEYYRGRNNLTAPVSRTIFHDITRLHLDDGLINCAKYFINYFFYKFGLETCFLMSVNVIGQRMDFYAMIHACWLIAVLYRRRRKAIAEIWPKYCCFLACIITFQYF   1206
           ****************** ********************** ************************************ ************ *****************

                                                  TM 24
mPiezo2   VCIGIPPAPCRDYPWRFKGAYFNDNIIKWLYFPDFIVRPNPVFLVYDFMLLLCASLQRQIFEDENKAAVRIMAGDNVEICMNLDAASFSQHNPVPDFIHCRSYLDMSKVI   1319
hPiezo2   ICIGIPPAPCRDYPWRFKGASFNDNIIKWLYFPDFIVRPNPVFLVYDFMLLLCASLQRQIFEDENKAAVRIMAGDNVEICMNLDAASFSQHNPVPDFIHCRSYLDMSKVI   1316
           ******************* *****************************************************************************************

               TM 25              TM 26                 TM 27
mPiezo2   IFSYLFWFVLTIIFITGTTRISIFCMGYLVACFYFLLFGGDLLLKPIKSILRYWDWLIAYNVFVITMKNILSIGACGYIGALVRNSCWLIQAFSLACTVKGYQMPEDDSR   1429
hPiezo2   IFSYLFWFVLTIIFITGTTRISIFCMGYLVACFYFLLFGGDLLLKPIKSILRYWDWLIAYNVFVITMKNILSIGACGYIGTLVHNSCWLIQAFSLACTVKGYQMPAANSP   1426
          ******************************************************************************** ** *********************   *

                     TM 28             Beam
mPiezo2   CKLPSGEAGIIWDSICFAFLLLQRRVFMSYYFLHVVADIKASQILASRGAELFQATIVKAVKARIEEEKKSMDQLKRQMDRIKARQQKYKKGKERMLSLTQESGEGQDIQ   1539
hPiezo2   CTLPSGEAGIIWDSICFAFLLLQRRVFMSYYFLHVVADIKASQILASRGAELFQATIVKAVKARIEEEKKSMDQLKRQMDRIKARQQKYKKGKERMLSLTQEPGEGQDMQ   1536
          * **************************************************************************************************** ***** *

                         Exon 33                                                         Exon 35
mPiezo2   KVSEEDDEREADKQKAKGKKKQWWRPWVDHASMVRSGDYYLFETDSEEEEEEELKKEDEEPPRKSAFQRAIGKFASAILALPKSVIKLPKTILQYLIRAAKFVYQAWITD   1649
hPiezo2   KLSEEDDEREADKQKAKGKKKQWWRPWVDHASMVRSGDYYLFETDSEEEEEEELKKEDEEPPRRSAFQRAIGKFASAILALPKSVIKLPKTILQYLIRAAKFVYQAWITD   1646
          * ************************************************************* **********************************************
```

bioRxiv preprint doi: https://doi.org/10.64898/2026.02.16.706133; this version posted February 17, 2026. The copyright holder for this preprint (which was not certified by peer review) is the author/funder, who has granted bioRxiv a license to display the preprint in perpetuity. It is made available under aCC-BY 4.0 International license.

```
mPiezo2  PKTALRQRRKEKKKLAREEQKERRKGSGDGPVEWEDREDEPVKKKSDGPDNIIKRIFNILKFTWVLFLATVDSFTTWLNSISREHIDISTVLRIERCMLTREIKKGNVPT  1759
hPiezo2  PKTALRQRHKEKKRSAREERKRRRKGSKEGPVEWEDREDEPIKKKSDGPDNIIKRIFNILKFTWVLFLATVDSFTTWLNSISREHIDISTVLRIERCMLTREIKKGNVPT  1756
         ******** ****  **** * *****  ************ ********************************************************************

                                                                                    Exon 40
mPiezo2  RESIHMYYQNHIMNLSRESGLDTIDEHSGAGSRAQAAHRMDSLDSRDSISSCYTEATLLISRQSTLDDLDGQDPVPKTSERARPRLRKMFSLDMSSSSADSGSVASSEPT  1869
hPiezo2  RESIHMYYQNHIMNLSRESGLDTIDEHPGAASGAQTAHRMDSLDSHDSI-SCYTEATMLFSRQSTLDDLDGQE-IPKTSERARPRLRKMLSMDMSSSSADSGSLASSEPT  1864
         *************************** ** * ** ********* *** ******* * ************   ************** * *********** ******

mPiezo2  QCTMLYSRQGTTETIEEVEAEAEEEVVEGLEPELHDAEEKEYAA--EYEAGVEEISLTPDEELPQFSTD--DCEAPPSYSKAVSFEHLSFASQDDSGAKNHMVVSPDDSR  1975
hPiezo2  QCTMLYSRQGTTETIEEVEAEQEEEAG-STAPEPREAKEYEATGYDVGAMGAEEASLTPEEELTQFSTLDGDVEAPPSYSKAVSFEHLSFGSQDDSAGKNRMAVSPDDSR  1973
         ********************* ***      **   * * *         * ** **** *** ****   * ***************** *****  ** * *******

                                                                    TM 29                 TM 30            TM 31
mPiezo2  TDKLESSILPPLTHELTASDLLMSKMFHDDELEESEKFYVDQPRFLLLFYAMYNTLVARSEMVCYFVIILNHMTSASIITLLLPILIFLWAMLSVPRPSRRFWMMAIVYT  2085
hPiezo2  TDKLGSSILPPLTHELTASELLLKKMFHDDELEESEKFYVGQPRFLLLFYAMYNTLVARSEMVCYFVIILNHMVSASMITLLLPILIFLWAMLSVPRPSRRFWMMAIVYT  2083
         **** ************** **  **************** ******************************** *** ********************************

                                                     TM 32
mPiezo2  EVAIVVKYFFQFGFFPWNKDLEIYKERPYFPPNIIGVEKKEGYVLYDLIQLLALFFHRSILKCHGLWDEDDIVDSNTDKEGSDDELSLDQGRRGSSDSLKSINLAASVES  2195
hPiezo2  EVAIVVKYFFQFGFFPWNKNVEVNKDKPYHPPNIIGVEKKEGYVLYDLIQLLALFFHRSILKCHGLWDEDDMTESGMAREESDDELSLGHGRRDSSDSLKSINLAASVES  2193
         *******************  *  *  ** *****************************************   *    * *******  *** ****************

mPiezo2  VHVTFPEQPAAIRRKRSCSSSQISPRSSFSSNRSKRGSTSTRNSSQKGSSVLSLKQKSKRELYMEKLQEHLIKAKAFTIKKTLQIYVPIRQFFYDLIHPDYSAVTDVYVL  2305
hPiezo2  VHVTFPEQQTAVRRKRSGSSSEPSQRSSFSSNRSQRGSTSTRNSSQKGSSVLSIKQKGKRELYMEKLQEHLIKAKAFTIKKTLEIYVPIKQFFYNLIHPEYSAVTDVYVL  2303
         ********  * ***** ***  * ********* ****************** *** ************************* ***** **** **** **********

         TM 33                                  TM 34                    TM 35                           TM 36
mPiezo2  MFLADTVDFIIIVFGFWAFGKHSAAADITSSLSEDQVPGPFLVMVLIQFGTMVVDRALYLRKTVLGKVIFQVILVFGIHFWMFFILPGVTERKFSQNLVAQLWYFVKCVY  2415
hPiezo2  MFLADTVDFIIIVFGFWAFGKHSAAADITSSLSEDQVPGPFLVMVLIQFGTMVVDRALYLRKTVLGKVIFQVILVFGIHFWMFFILPGVTERKFSQNLVAQLWYFVKCVY  2413
         **************************************************************************************************************

                                  Anchor                                                                Outer Pore Helix
mPiezo2  FGLSAYQIRCGYPTRVLGNFLTKSYNYVNLFLFQGFRLVPFLTELRAVMDWVWTDTTLSLSSWICVEDIYAHIFILKCWRESEKRYPQPRGQKKKKAVKYGMGGMIIVLL  2525
hPiezo2  FGLSAYQIRCGYPTRVLGNFLTKSYNYVNLFLFQGFRLVPFLTELRAVMDWVWTDTTLSLSSWICVEDIYAHIFILKCWRESEKRYPQPRGQKKKKVVKYGMGGMIIVLL  2523
         ************************************************************************************************ *************

                           Cap
mPiezo2  ICIVWFPLLFMSLIKSVAGVINQPLDVSVTITLGGYQPIFTMSAQQSQLKVMDNSKYNEFLKSFGPNSGAMQFLENYEREDVTVAELEGNSNSLWTISPPSKQKMIQELT  2635
hPiezo2  ICIVWFPLLFMSLIKSVAGVINQPLDVSVTITLGGYQPIFTMSAQQSQLKVMDQQSFNKFIQAFSRDTGAMQFLENYEKEDITVAELEGNSNSLWTISPPSKQKMIHELL  2633
         *****************************************************    * *   *    ********** ** ************************ ** 

mPiezo2  DPNSCFSVVFSWSIQRNMTLGAKAEIATDKLSFPLAVATRNSIAKMIAGNDTESSNTPVTIEKIYPYYVKAPSDSNSKPIKQLLSENNFMNITIILFRDNVTKSNSEWWV  2745
hPiezo2  DPNSSFSVVFSWSIQRNLSLGAKSEIATDKLSFPLKNITRKNIAKMIAGNSTESSKTPVTIEKIYPYYVKAPSDSNSKPIKQLLSENNFMDITIILSRDNTTKYNSEWWV  2743
         **** ************  **** ***********   **  ******** **** ********************************** ***** *** ** ******

                                              Inner Pore Helix                  C-Terminal Domain
mPiezo2  LNLTGSRIFNQGSQALELVVFNDKVSPPSLGFLAGYGIMGLYASVVLVIGKFVREFFSGISHSIMFEELPNVDRILKLCTDIFLVRETGELELEEDLYAKLIFLYRSPET  2855
hPiezo2  LNLTGNRIYNPNSQALELVVFNDKVSPPSLGFLAGYGIMGLYASVVLVIGKFVREFFSGISHSIMFEELPNVDRILKLCTDIFLVRETGELELEEDLYAKLIFLYRSPET  2853
         ***** ** *  **************************************************************************************************

mPiezo2  MIKWTREKTN  2865
hPiezo2  MIKWTREKTN  2863
         **********
```

Supplemental Figure 1. Sequence alignment and structural annotation of mouse and human Piezo2. *Sequence alignment of mouse and human Piezo2 with all exons present. Alternatively spliced exons are highlighted by boxes, main structural features of mouse Piezo2 (PDB: 6KG7) are colored red, and domains that are not structurally resolved are shaded in grey.*

bioRxiv preprint doi: https://doi.org/10.64898/2026.02.16.706133; this version posted February 17, 2026. The copyright holder for this preprint (which was not certified by peer review) is the author/funder, who has granted bioRxiv a license to display the preprint in perpetuity. It is made available under aCC-BY 4.0 International license.

Supplemental Figure 2

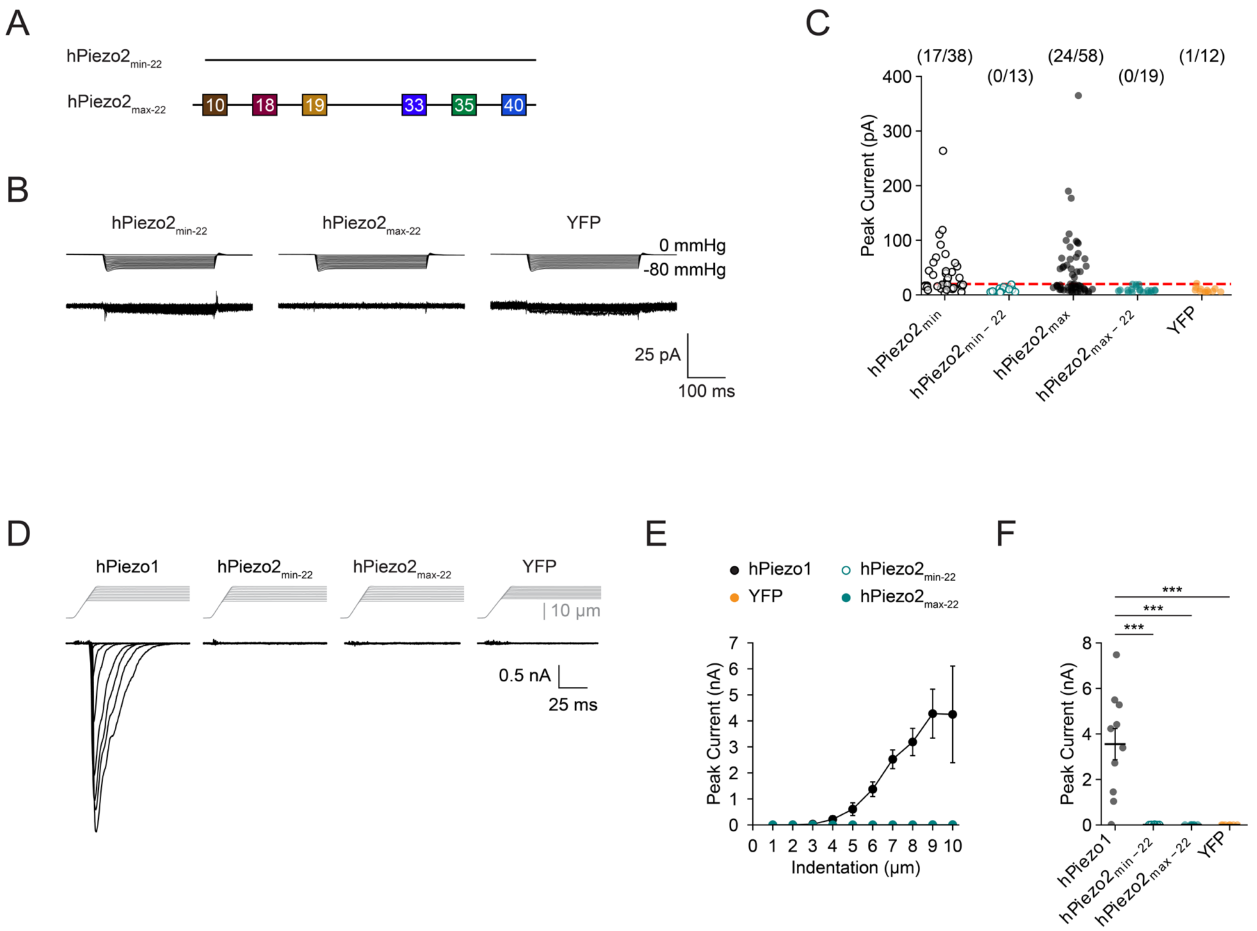

Supplemental Figure 2. Removal of exon 22 from hPiezo2$_{max}$ and hPiezo2$_{min}$.

A, *Schematic showing the alternatively spliced exons present in hPiezo2$_{min-22}$ and hPiezo2$_{max-22}$.* ***B***, *Top: Pressure-step protocol from 0 to -80 mmHg (Δ = -5 mmHg) and representative currents recorded from Neuro2A-Piezo1ko cells heterologously expressing hPiezo2$_{min-22,}$ hPiezo2$_{max-22}$, or YFP.* ***C***, *Peak current amplitudes obtained with the above protocol from cells expressing hPiezo2$_{min}$, hPiezo2$_{min-22}$, hPiezo2$_{max}$, hPiezo2$_{max-22}$, or YFP alone. The red dashed line illustrates the 20 pA threshold above which patches were used for analyzing tension responses. The number of patches with peak currents > 20 pA and the total number of patches are shown above.* ***D*** *Indentation-step protocol (Δ = 1 µm) and currents recorded from Neuro2A-Piezo1ko cells heterologously expressing hPiezo1, hPiezo2$_{min-22}$, hPiezo2$_{max-22}$, or YFP.* ***E***, *Mean*

bioRxiv preprint doi: https://doi.org/10.64898/2026.02.16.706133; this version posted February 17, 2026. The copyright holder for this preprint (which was not certified by peer review) is the author/funder, who has granted bioRxiv a license to display the preprint in perpetuity. It is made available under aCC-BY 4.0 International license.

indentation response curves for hPiezo1 (n = 10), hPiezo2$_{min-22}$ (n = 7), hPiezo2$_{max-22}$ (n = 7), and YFP (n = 7). Data are represented as mean±S.E.M. Peak current amplitude values for all individual patches shown in (D). Bars indicate the mean±S.E.M. ***F****, Mean values for peak current amplitude are as follows: hPiezo1 = 3,553±687 pA; hPiezo2$_{min-22}$ = 10±1 pA; hPiezo2$_{max-22}$ = 12±1 pA; YFP = 6±1 pA. Significance was determined using a one-way ANOVA (F = 16.2, $p < 0.005$) and Tukey's HSD post-hoc comparison (hPiezo1/hPiezo2$_{min-22}$, $p < 0.005$; hPiezo1/hPiezo2$_{max-22}$, $p < 0.005$; hPiezo1/YFP, $p < 0.005$).*

bioRxiv preprint doi: https://doi.org/10.64898/2026.02.16.706133; this version posted February 17, 2026. The copyright holder for this preprint (which was not certified by peer review) is the author/funder, who has granted bioRxiv a license to display the preprint in perpetuity. It is made available under aCC-BY 4.0 International license.

Supplemental Figure 3

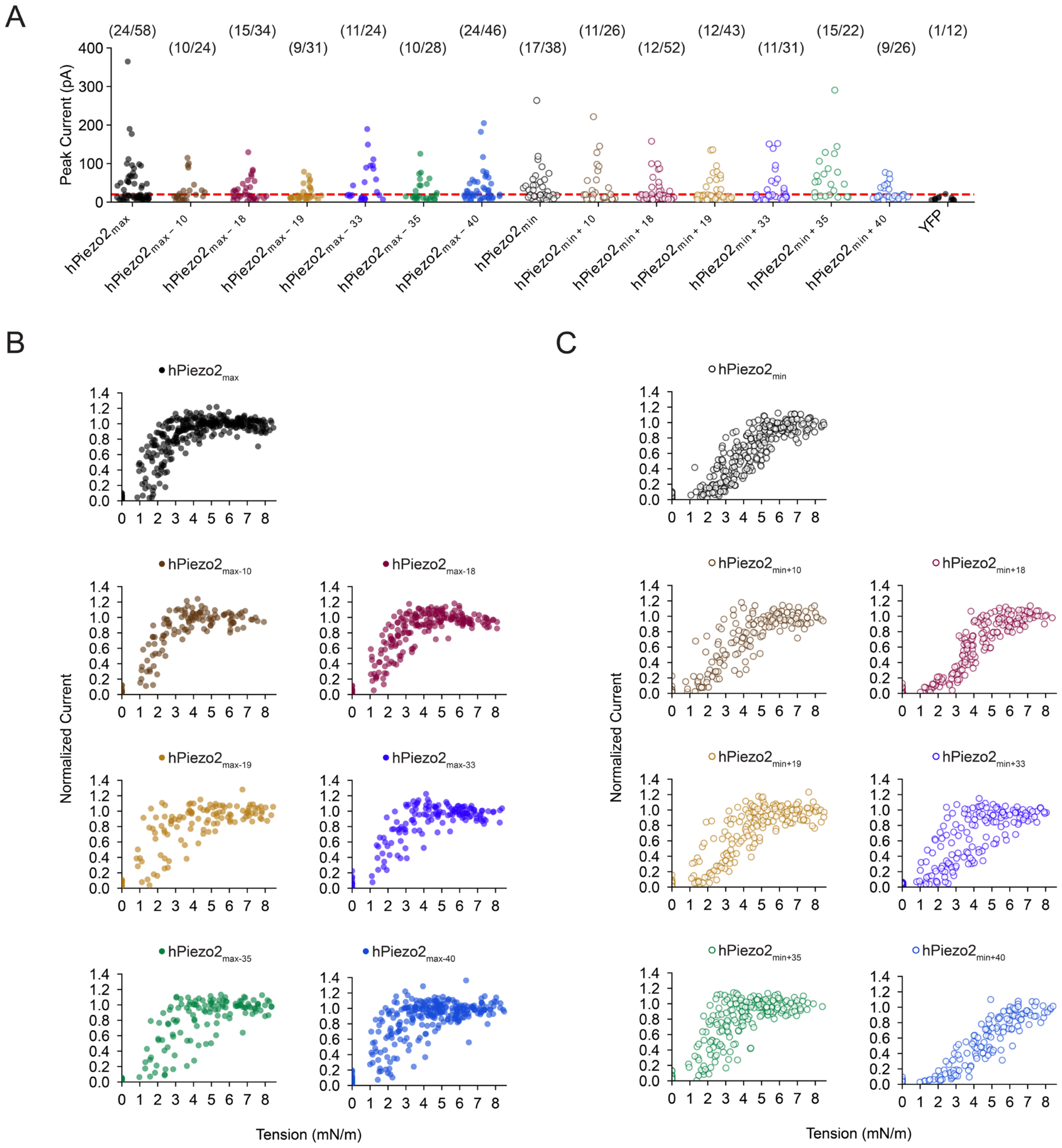

Supplemental Figure 3. Peak currents and individual tension responses for hPiezo2$_{max(-)}$ and hPiezo2$_{min(+)}$ constructs. ***A****, Peak current amplitudes obtained from Neuro2A-Piezo1ko cells heterologously expressing hPiezo2$_{max(-)}$ and hPiezo2$_{min(+)}$ constructs. The red dashed line illustrates the 20 pA threshold above which patches were used to calculate tension responses.*

bioRxiv preprint doi: https://doi.org/10.64898/2026.02.16.706133; this version posted February 17, 2026. The copyright holder for this preprint (which was not certified by peer review) is the author/funder, who has granted bioRxiv a license to display the preprint in perpetuity. It is made available under aCC-BY 4.0 International license.

The number of patches with a peak current > 20 pA and the total number of patches are shown above. ***B****, Normalized tension response for all hPiezo2$_{max(-)}$ constructs. Each point represents the tension and normalized current value elicited by one pressure step.* ***C****, Same as in (B), but for all hPiezo2$_{min(+)}$ constructs.*

bioRxiv preprint doi: https://doi.org/10.64898/2026.02.16.706133; this version posted February 17, 2026. The copyright holder for this preprint (which was not certified by peer review) is the author/funder, who has granted bioRxiv a license to display the preprint in perpetuity. It is made available under aCC-BY 4.0 International license.

Supplemental Figure 4

A

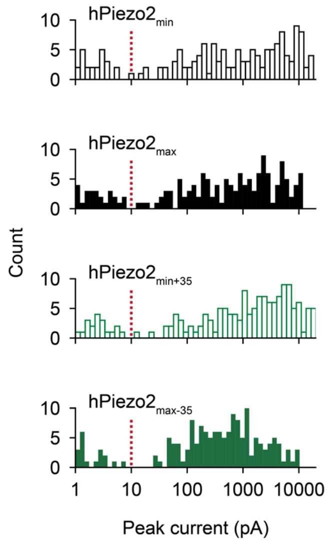

Supplemental Figure 4. Indentation-induced peak current histograms for hPiezo2$_{min}$, hPiezo2$_{max}$, hPiezo2$_{min+35}$, and hPiezo2$_{max-35}$. ***A***, *Histograms of peak currents from all indentation steps for each construct. The red dashed line illustrates the 10 pA peak current level, which was used as a response threshold.*

bioRxiv preprint doi: https://doi.org/10.64898/2026.02.16.706133; this version posted February 17, 2026. The copyright holder for this preprint (which was not certified by peer review) is the author/funder, who has granted bioRxiv a license to display the preprint in perpetuity. It is made available under aCC-BY 4.0 International license.

Supplemental Figure 5

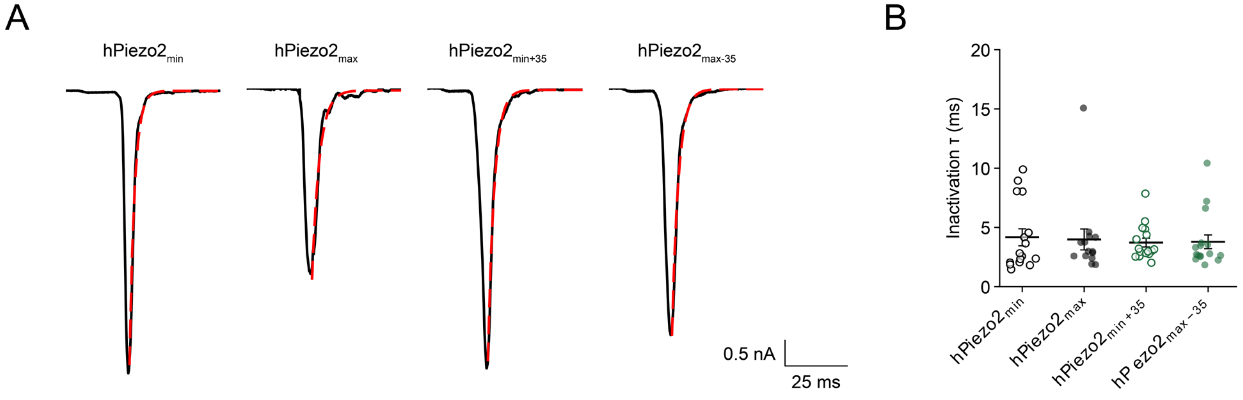

Supplemental Figure 5. Inactivation kinetics for hPiezo2$_{min}$, hPiezo2$_{max}$, hPiezo2$_{min+35}$, and hPiezo2$_{max-35}$. ***A***, *Representative currents recorded from final indentation step for a whole-cell poke protocol from Neuro2A-Piezo1ko cells heterologously expressing hPiezo2$_{min}$, hPiezo2$_{max}$, hPiezo2$_{min+35}$, or hPiezo2$_{max-35}$. Currents were fit with a single exponential (red) and their rates of inactivation (τ) are follows: hPiezo2$_{min}$ τ = 2.3±0.1 ms; hPiezo2$_{max}$ τ = 3.0±0.1 ms; hPiezo2$_{min+35}$ τ = 2.7±0.1 ms; hPiezo2$_{max-35}$ τ = 2.6±0.1 ms.* ***B***, *Inactivation time constants from single exponential fits to individual currents. Error bars indicate the mean±S.E.M. Mean values for inactivation (τ) are as follows: hPiezo2$_{min}$ τ = 4.2±0.7 ms; hPiezo2$_{max}$ τ = 4.0±0.9 ms; hPiezo2$_{min+35}$ τ = 3.7±0.4 ms; hPiezo2$_{max-35}$ τ = 3.8±0.6 ms. Significance was determined using a one-way ANOVA (F = 0.10, p = 0.96).*

bioRxiv preprint doi: https://doi.org/10.64898/2026.02.16.706133; this version posted February 17, 2026. The copyright holder for this preprint (which was not certified by peer review) is the author/funder, who has granted bioRxiv a license to display the preprint in perpetuity. It is made available under aCC-BY 4.0 International license.

Supplemental Figure 6

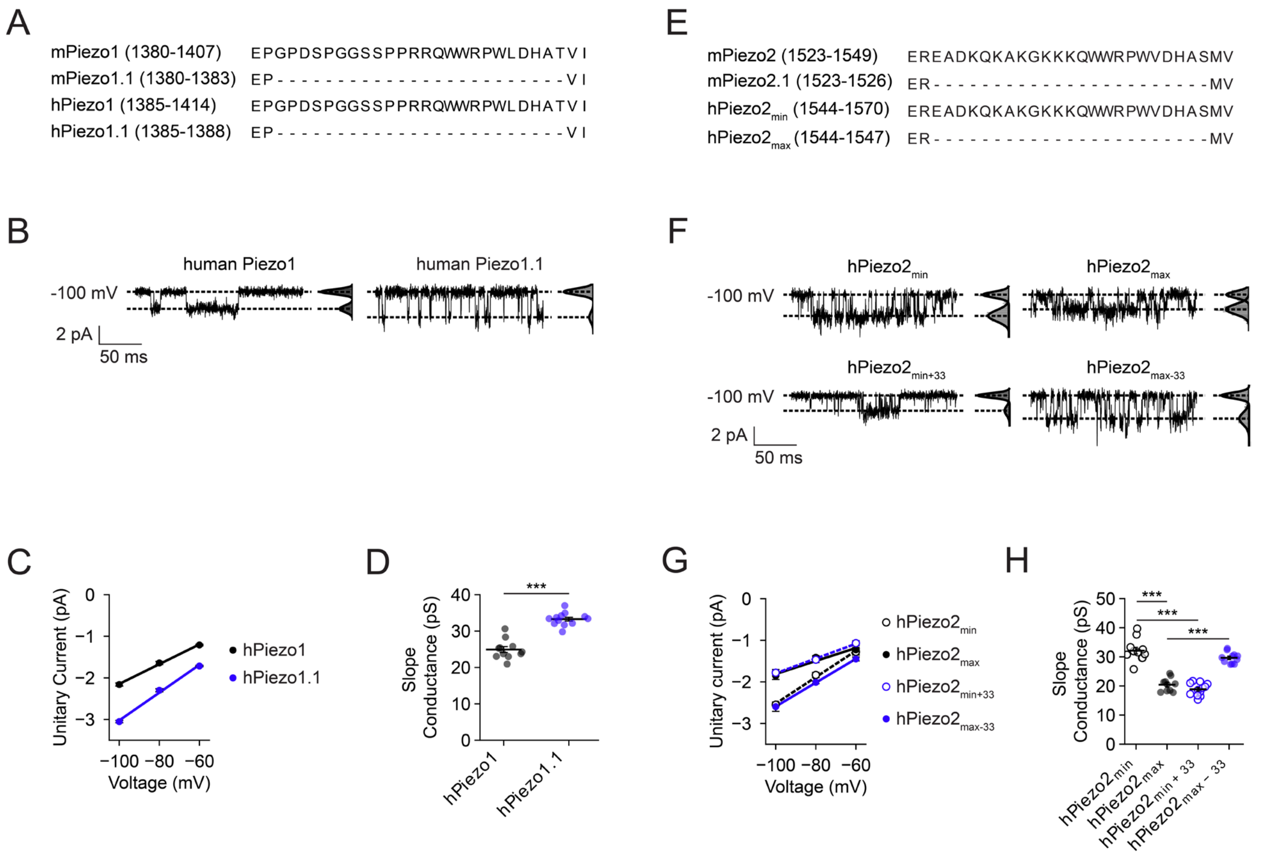

Supplemental Figure 6. Single channel conductances of hPiezo1 and hPiezo2 constructs. ***A***, *Sequence alignment of mouse and human Piezo1 and Piezo1.1 that contain and lack exon 30, respectively.* ***B***, *Representative cell-attached recordings from Neuro2A-Piezo1ko cells heterologously expressing hPiezo1 or hPiezo1.1 at a holding potential of -100mV and their current amplitude histograms.* ***C***, *Representative current-voltage relationships generated from Neuro2A-Piezo1ko cells expressing hPiezo1 and hPiezo1.1. Slope conductances are g = 23.8±1.0 pS (hPiezo1) and g = 33.4±0.8 pS (hPiezo1.1).* ***D***, *Slope conductances from all individual patches for hPiezo1 (n = 11) and hPiezo1.1 (n = 12). Error bars indicate mean±S.E.M. Mean slope conductances are as follows: hPiezo1 g = 24.9±0.8 pS; hPiezo1.1 g = 33.2±0.5 pS. Significance was determined using Welch's unpaired t-test (t = 8.7, p < 0.0005).* ***E***, *Sequence alignment of mouse Piezo2, mouse Piezo2.1., human Piezo2$_{min}$, and human Piezo2$_{max}$ that*

bioRxiv preprint doi: https://doi.org/10.64898/2026.02.16.706133; this version posted February 17, 2026. The copyright holder for this preprint (which was not certified by peer review) is the author/funder, who has granted bioRxiv a license to display the preprint in perpetuity. It is made available under aCC-BY 4.0 International license.

contain and lack exon 33, respectively. ***F***, *Representative cell-attached recordings from Neuro2A-Piezo1ko cells heterologously expressing hPiezo2$_{min}$, hPiezo2$_{max}$, Piezo2$_{min+33}$, or Piezo2$_{max-33}$, at a holding potential of -100mV and their current amplitude histograms.* ***G***, *Representative current-voltage relationships generated from Neuro2A-Piezo1ko cells expressing hPiezo2$_{min}$, hPiezo2$_{max}$, Piezo2$_{min+33}$, and Piezo2$_{max-33}$. Slope conductances are as follows: hPiezo2$_{min}$ g = 31.7±1.9 pS; hPiezo2$_{max}$ g = 15.7±2.5 pS; Piezo2$_{min+33}$ g = 17.8±2.3 pS; Piezo2$_{max-33}$ g = 28.7±3.1 pS.* ***H***, *Slope conductance values from all individual patches for hPiezo2$_{min}$ (n =11), hPiezo2$_{max}$ (n = 10), Piezo2$_{min+33}$ (n = 12), and Piezo2$_{max-33}$ (n = 11). Error bars indicate mean±S.E.M. Mean slope conductances are as follows: hPiezo2$_{min}$ g = 32.1±1.2 pS; hPiezo2$_{max}$ g = 20.4±0.7 pS; hPiezo2$_{min+33}$ g = 18.9±0.6 pS; hPiezo2$_{max-33}$ g = 29.7±0.6 pS. Significance was determined using a one-way ANOVA (F = 68.8, $p < 0.0005$) and Tukey's HSD post-hoc comparison (hPiezo2$_{max}$/hPiezo2$_{min}$: $p < 0.0005$; hPiezo2$_{max}$/hPiezo2$_{max-33}$: $p < 0.0005$; hPiezo2$_{min}$/hPiezo2$_{min+33}$: $p < 0.0005$).*

bioRxiv preprint doi: https://doi.org/10.64898/2026.02.16.706133; this version posted February 17, 2026. The copyright holder for this preprint (which was not certified by peer review) is the author/funder, who has granted bioRxiv a license to display the preprint in perpetuity. It is made available under aCC-BY 4.0 International license.

Supplemental Figure 7

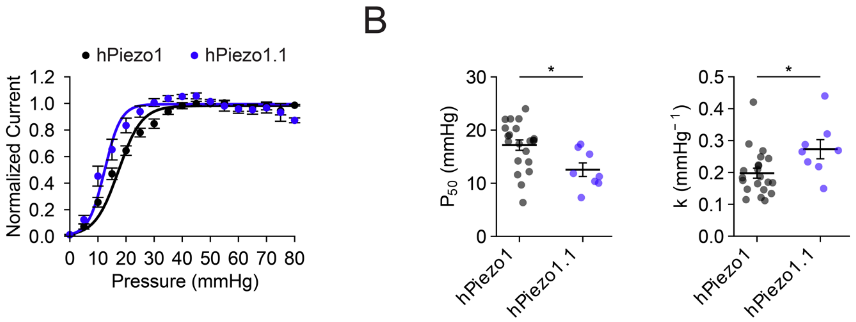

Supplemental Figure 7. Pressure responses of hPiezo1 and hPiezo1.1. ***A***, *Average pressure response curves of hPiezo1 (n = 21) and hPiezo1.1 (n = 8). Data are plotted as normalized mean±S.E.M.* ***B***, *Values of pressure of half-maximal activation (P_{50}) and slope (k) from all individual patches. Error bars indicate mean±S.E.M. Mean values for P_{50} and k are as follows: hPiezo1 P_{50} = 17.2±1.0 mmHg, k = 0.20±0.02 mmHg^{-1}; hPiezo1.1 P_{50} = 12.5±1.3 mmHg, k = 0.27±0.03 mmHg^{-1}. Significance was determined using Welch's unpaired t-test (P_{50} t = 2.9, p = 0.011; k t = 2.23, p = 0.048).*

bioRxiv preprint doi: https://doi.org/10.64898/2026.02.16.706133; this version posted February 17, 2026. The copyright holder for this preprint (which was not certified by peer review) is the author/funder, who has granted bioRxiv a license to display the preprint in perpetuity. It is made available under aCC-BY 4.0 International license.

Supplemental Figure 8

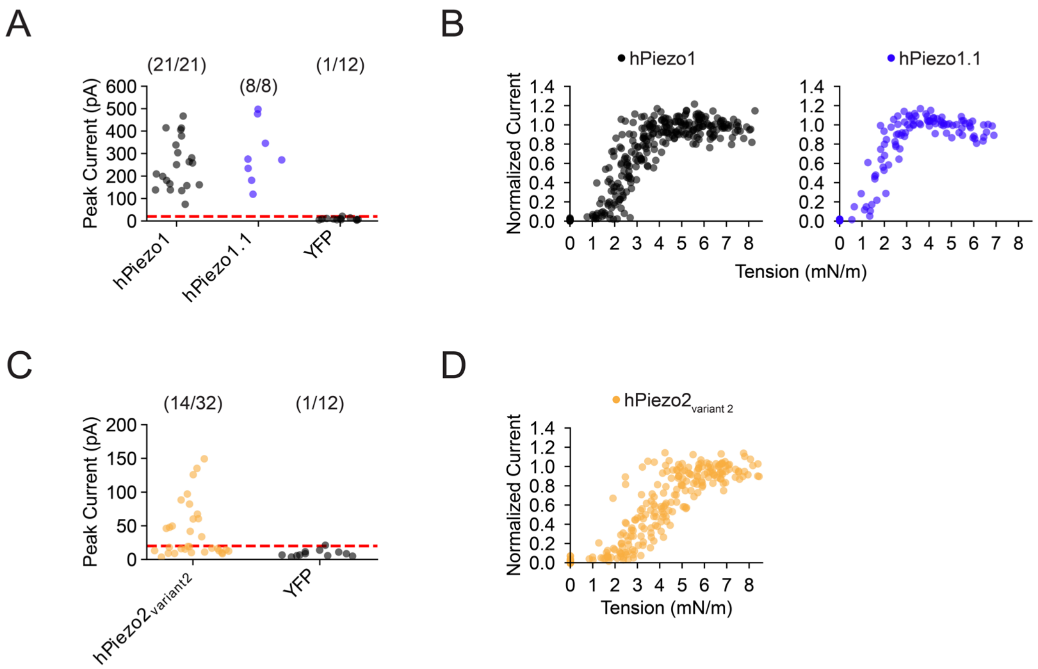

Supplemental Figure 8. Peak currents and individual tension-responses of hPiezo1, hPiezo1.1, and hPiezo2 Variant 2. ***A***, *Peak current amplitudes from Neuro2A-Piezo1ko cells heterologously expressing hPiezo1 and hPiezo1.1. The red dashed line illustrates the 20 pA threshold above which patches were used for analyzing tension responses. The number of patches with a peak current > 20 pA and the total number of patches are shown above.* ***B***, *Normalized tension responses. Each point represents the tension and normalized current value elicited by one pressure step.* ***C***, *Same as in (A) but for hPiezo2 variant 2.* ***D***, *Same as in (B) but for hPiezo2 variant 2.*

bioRxiv preprint doi: https://doi.org/10.64898/2026.02.16.706133; this version posted February 17, 2026. The copyright holder for this preprint (which was not certified by peer review) is the author/funder, who has granted bioRxiv a license to display the preprint in perpetuity. It is made available under aCC-BY 4.0 International license.

Table S1: Primers for generating hPiezo2max starting from hPiezo2variant2 (hPIEZO2-pIRES2-mCherry-WPRE).

Starting construct number/name (included exons)	Exon added/removed	Unique primer number	Primer	Generated construct number/name (included exons)
#1<br>hPiezo2$_{variant2}$ (10, 22, 33)	+18	1 (forward)	5'-ggtttacctgataataaatctggcgcaccccgagggc-3'	#2<br>hPiezo2 (10, 22, 18, 33)
		2 (reverse)	5'-cgcccatttacctttgcatgacgataaatggtgttgtcctccttgcttggg-3'	
#2<br>hPiezo2 (10, 22, 18, 33)	+19	3 (forward)	5'-gcctatacatcaaaatctggcgcaccccgagggc-3'	#3<br>hPiezo2 (10, 22, 18, 19, 33)
		4 (reverse)	5'-aacttcttctttatagaatttattatcaggtaaacccgcccatttacctttgcatg-3'	
#3<br>hPiezo2 (10, 22, 18, 19, 33)	+35 part 1	5 (forward)	5'-aatcctcgctctgccaaagtcagttttcgtgtaccaagcctgg-3'	#4<br>hPiezo2 (10, 22, 18, 19, 33, 35 part 1)
		6 (reverse)	5'-gcggatgcaaacttccctatggcccgctgaaaggcgcttcttctag-3'	
#4<br>hPiezo2 (10, 22, 18, 19, 33, 35 part 1)	+35 part 2	7 (forward)	5'-cagtacctgattagagccgcaaaattcgtgtaccaagcctgg-3'	#5<br>hPiezo2$_{max-40}$ (10, 22, 18, 19, 33, 35)
		8 (reverse)	5'-cagtatggttttaggcagctttataactgactttggcagagc-3'	
#5<br>hPiezo2$_{max-40}$ (10, 22, 18, 19, 33, 35)	+40 part 1	9 (forward)	5'-agtcggcaatcaactttggatgatctcagcgaaccaacacaatgc-3'	#6<br>hPiezo2 (10, 22, 18, 19, 33, 35, 40 part 1)
		10 (reverse)	5'-gaagagcatagtggcctcggtataacagctgatgctgtcgtggct-3'	
#6	+40 part 2	11 (forward)	5'-gagcgggctcggcccagactgcggaagagcgaaccaacacaatgc-3'	#7

bioRxiv preprint doi: https://doi.org/10.64898/2026.02.16.706133; this version posted February 17, 2026. The copyright holder for this preprint (which was not certified by peer review) is the author/funder, who has granted bioRxiv a license to display the preprint in perpetuity. It is made available under aCC-BY 4.0 International license.

hPiezo2 (10, 22, 18, 19, 33, 35, 40 part 1)		12 (reverse)	5'-actggttttaggaatttcctgaccgtcgagatcatccaaagttgattgc-3'	hPiezo2 (10, 22, 18, 19, 33, 35, 40 part 1+2)
#7<br><br>hPiezo2 (10, 22, 18, 19, 33, 35, 40 part 1+2)	+40 part 3	13 (forward)	5'-tcagcagatagcgggagtctcgcctctagcgaaccaacacaatgcacc-3'	#8<br><br>hPiezo2$_{max}$ (10, 18, 19, 22, 33, 35, 40)
		14 (reverse)	5'-tgaggaagacatatccattgagagcatcttccgcagtctgggccg-3'	

hPiezo2$_{max}$ was generated by sequentially adding missing spliced exons to hPiezo2$_{variant2}$. Newly generated constructs whose tension sensitivity was measured are indicated in red. Exons too large for insertion in a single cloning step were assembled by sequential addition of exon fragments.

bioRxiv preprint doi: https://doi.org/10.64898/2026.02.16.706133; this version posted February 17, 2026. The copyright holder for this preprint (which was not certified by peer review) is the author/funder, who has granted bioRxiv a license to display the preprint in perpetuity. It is made available under aCC-BY 4.0 International license.

Table S2: Primers for generating hPiezo2$_{min}$ and hPiezo2$_{min-22}$, starting from hPiezo2$_{variant2}$ (hPIEZO2-pIRES2-mCherry-WPRE).

Starting construct number/name (included exons)	Exon added/removed	Unique primer number	Primer	Generated construct number/name (included exons)
#1<br>hPiezo2$_{variant2}$<br>(10, 22, 33)	-33	15 (forward)	5'-atggtgagaagcggc gac-3'	#9<br>hPiezo2$_{min+10}$<br>(10, 22)
		16 (reverse)	5'-tctctcgtcatcttcctc actc-3'	
#9<br>hPiezo2$_{min+10}$<br>(10, 22)	-10	17 (forward)	5'-gggctcctggtgacc gtg-3'	#10<br>hPiezo2$_{min}$<br>(22)
		18 (reverse)	5'-ctttctctcgtcggtgg gg-3'	
#10<br>hPiezo2$_{min}$<br>(22)	-22	19 (forward)	5'-cccaacgagaatcaga cc-3'	#11<br>hPiezo2$_{min-22}$<br>(all exons removed)
		20 (reverse)	5'-ctctttcacgctcaccca-3'	

hPiezo2$_{min}$ and hPiezo2$_{min-22}$ were generated by sequentially removing the remaining spliced exons from hPiezo2$_{variant2}$.

bioRxiv preprint doi: https://doi.org/10.64898/2026.02.16.706133; this version posted February 17, 2026. The copyright holder for this preprint (which was not certified by peer review) is the author/funder, who has granted bioRxiv a license to display the preprint in perpetuity. It is made available under aCC-BY 4.0 International license.

Table S3: Primers for generating additional hPiezo2$_{max(-)}$ constructs, starting from hPiezo2$_{max}$.

Starting construct number/name (included exons)	Exon added/removed	Unique primer number	Primer	Generated construct number/name (included exons)
#8 hPiezo2$_{max}$ (10, 18, 19, 22, 33, 35, 40)	-10	17 (forward)	5’-gggctcctggtgaccgtg-3’	#12 hPiezo2$_{max-10}$ (18, 19, 22, 33, 35, 40)
		18 (reverse)	5’-ctttctctcgtcggtgggg-3’	
#8 hPiezo2$_{max}$ (10, 18, 19, 22, 33, 35, 40)	-18	21 (forward)	5’-tctataaagaagaagttgcc-3’	#13 hPiezo2$_{max-18}$ (10, 19, 22, 33, 35, 40)
		22 (reverse)	5’-acgataaatggtgttgtc-3’	
#8 hPiezo2$_{max}$ (10, 18, 19, 22, 33, 35, 40)	-19	23 (forward)	5’-ctggcgcaccccgagggc-3’	#14 hPiezo2$_{max-19}$ (10, 18, 22, 33, 35, 40)
		24 (reverse)	5’-atttattatcaggtaaacccgcccatttacctttgcatg-3’	
#8 hPiezo2$_{max}$ (10, 18, 19, 22, 33, 35, 40)	-22	19 (forward)	5’-cccaacgagaatcagacc-3’	#15 hPiezo2$_{max-22}$ (10, 18, 19, 33, 35, 40)
		20 (reverse)	5’-ctctttcacgctcaccca-3’	
#8 hPiezo2$_{max}$ (10, 18, 19, 22, 33, 35, 40)	-33	15 (forward)	5’-atggtgagaagcggcgac-3’	#16 hPiezo2$_{max-33}$ (10, 18, 19, 22, 35, 40)
		16 (reverse)	5’-tctctcgtcatcttcctcactc-3’	
#8 hPiezo2$_{max}$ (10, 18, 19, 22, 33, 35, 40)	-35	25 (forward)	5’-ttcgtgtaccaagcctgg-3’	#17 hPiezo2$_{max-35}$ (10, 18, 19, 22, 33, 35, 40)
		25 (forward)	5’-ttcgtgtaccaagcctgg-3’	

hPiezo2$_{max(-)}$ constructs were generated by removing individual spliced exons from hPiezo2$_{max}$.

bioRxiv preprint doi: https://doi.org/10.64898/2026.02.16.706133; this version posted February 17, 2026. The copyright holder for this preprint (which was not certified by peer review) is the author/funder, who has granted bioRxiv a license to display the preprint in perpetuity. It is made available under aCC-BY 4.0 International license.

Table S4: Primers for generating additional hPiezo2$_{min(+)}$ constructs, starting from hPiezo2$_{min}$.

Starting construct number/name (included exons)	Exon added/removed	Unique primer number	Primer	Generated construct number/name (included exons)
#10<br>hPiezo2$_{min}$ (22)	+18	1 (forward)	5’-ggtttacctgataataaatctggcgcaccccgagggc-3’	#18<br>hPiezo2$_{min+18}$ (18, 22)
		2 (reverse)	5’-cgcccatttacctttgcatgacgataaatggtgttgtcctccttgcttggg-3’	
#10<br>hPiezo2$_{min}$ (22)	+19	27 (forward)	5’-gcctatacatcaaaatctggcgcaccccgagggc-3’	#19<br>hPiezo2$_{min+19}$ (19, 22)
		28 (reverse)	5’-aacttcttctttatagaacgataaatggtgttgtcctccttgcttggg-3’	
#10<br>hPiezo2$_{min}$ (22)	+35 part 1	5 (forward)	5’-aatcctcgctctgccaaagtcagttttcgtgtaccaagcctgg-3’	#20<br>hPiezo2 (22, 35 part 1)
		6 (reverse)	5’-gcggatgcaaacttccctatggcccgctgaaaggcgcttcttctag-3’	
#20<br>hPiezo2 (22, 35 part 1)	+35 part 2	7 (forward)	5’-cagtacctgattagagccgcaaaattcgtgtaccaagcctgg-3’	#21<br>hPiezo2$_{min+35}$ (22, 35)
		8 (reverse)	5’-cagtatggttttaggcagctttataactgactttggcagagc-3’	
#10<br>hPiezo2$_{min}$ (22)	+40 part 1	9 (forward)	5’-agtcggcaatcaactttggatgatctcagcgaaccaacacaatgc-3’	#22<br>hPiezo2 (22, 35, 40 part 1)
		10 (reverse)	5’-gaagagcatagtggcctcggtataacagctgatgctgtcgtggct-3’	
#22<br>hPiezo2 (22, 35, 40 part 1)	+40 part 2	11 (forward)	5’-gagcgggctcggcccagactgcggaagagcgaaccaacacaatgc-3’	#23<br>hPiezo2 (22, 35, 40 part 1+2)
		12 (reverse)	5’-actggttttaggaatttcctgaccgtcgagatcatccaaagttgattgc-3’	
#23	+40 part 3	13 (forward)	5’-tcagcagatagcgggagtctcgcctctagcgaaccaacacaatgcacc-3’	#24

bioRxiv preprint doi: https://doi.org/10.64898/2026.02.16.706133; this version posted February 17, 2026. The copyright holder for this preprint (which was not certified by peer review) is the author/funder, who has granted bioRxiv a license to display the preprint in perpetuity. It is made available under aCC-BY 4.0 International license.

hPiezo2 (22, 35, 40 part 1+2)		14 (reverse)	5'-tgaggaagacatatccattg agagcatcttccgcagtctggg ccg-3'	hPiezo2$_{min+40}$ (22, 40)
#1<br>hPiezo2$_{variant2}$ (10, 22, 33)	-10	17 (forward)	5'-gggctcctggtgaccgtg-3'	#25<br>hPiezo2$_{min+33}$ (22, 33)
		18 (reverse)	5'-ctttctctcgtcggtgggg-3'	

hPiezo2$_{min(+)}$ constructs were generated by adding individual spliced exons to hPiezo2$_{min}$ or removing individual spliced exons from hPiezo2$_{variant2}$. Exons too large for insertion in a single cloning step were assembled by sequential addition of exon fragments.

bioRxiv preprint doi: https://doi.org/10.64898/2026.02.16.706133; this version posted February 17, 2026. The copyright holder for this preprint (which was not certified by peer review) is the author/funder, who has granted bioRxiv a license to display the preprint in perpetuity. It is made available under aCC-BY 4.0 International license.

Table S5: Primers for generating hPiezo1, starting from hPiezo1 (Human Piezo1-pIRES2-EGFP).

Starting construct number/name (included exons)	Exon added/removed	Unique primer number	Primer	Generated construct number/name (included exons)
#26<br><br>hPiezo1<br>(30)	-30	29<br>(forward)	5'-gtcatccactccggggacta cttcc-3'	#27<br><br>hPiezo1.1<br>(all exons removed)
		30<br>(reverse)	5'-tggctccaggccgggg tc-3'	

hPiezo2$_{min(+)}$ constructs were generated by adding individual spliced exons to hPiezo2$_{min}$. Exons too large for insertion in a single cloning step were assembled by sequential addition of exon fragments.
